# Complications 18 years after polyacrylamide hydrogel augmentation mammoplasty: a case report and histopathological analysis

**DOI:** 10.1093/jscr/rjab276

**Published:** 2021-06-22

**Authors:** Matthew DeLuca, Alexandra Shapiro, Elliot Banayan, Gregory Zielinski, Irena Karanetz, Armand Asarian, Philip Xiao

**Affiliations:** St. George’s University School of Medicine, True Blue, Grenada, WI, USA; St. George’s University School of Medicine, True Blue, Grenada, WI, USA; Department of Surgery, The Brooklyn Hospital Center, Brooklyn, NY 11201, USA; Department of Surgery, The Brooklyn Hospital Center, Brooklyn, NY 11201, USA; Department of Surgery, The Brooklyn Hospital Center, Brooklyn, NY 11201, USA; Department of Surgery, The Brooklyn Hospital Center, Brooklyn, NY 11201, USA; Department of Pathology, The Brooklyn Hospital Center, Brooklyn, NY 11201, USA

## Abstract

Polyacrylamide hydrogel (PAAG) is a synthetic substance previously used as an injectable material for augmentation mammoplasty. Current literature has demonstrated that the average time from PAAG injection to the onset of complication ranges from 6 to 39 months. We present a unique case report describing the onset of complications 18 years after PAAG augmentation mammoplasty. To the best of our knowledge, the presentation of a healthy female who experienced unprovoked expansion of breast tissue >15 years after polyacrylamide injection has not been previously reported in surgical literature. This suggests that serious complications of PAAG injection may occur later than the literature has previously described. Importantly, this case is the first demonstration of the successful surgical removal of polyacrylamide 18 years after injection. Additionally, this case also provides a histopathological analysis of breast capsules which showed evidence of an extensive chronic inflammatory reaction to polyacrylamide, consistent with previous reports.

## INTRODUCTION

PAAG is a gelatin-like synthetic substance with a purple hue which contains ~2.5% cross-linked polyacrylamide and 97.5% water [1]. PAAG has been used as an injectable material for cosmetic procedures, particularly augmentation mammoplasty [1]. Although the technique itself is minimally invasive and may achieve desired increases in breast size, use of PAAG is associated with harmful effects and high rates of complications, including tissue expansion, pain, infection, and breast cancer [2]. The largest published case series describing PAAG-related complications demonstrated that the average time course from PAAG injection to complication is 6–39 months [3]. Our case suggests that serious complications of PAAG injection which require surgical intervention may occur much later than the literature has previously described. Although several large-scale studies have proposed guidelines contributing to the standardization in surgical management of these complications, there is a paucity of detailed reports demonstrating a successful surgical approach toward the removal of polyacrylamide more than a decade after its initial injection.

## CASE REPORT

A 46-year-old otherwise healthy female presented to the clinic with complaints of progressive breast enlargement and pain over the past 3 months. Specifically, she described a more pronounced acutely developing enlargement of the right breast when compared to the left, associated with worsening discomfort and pain. Of note, the patient underwent bilateral augmentation mammoplasty using PAAG injections in China 18 years ago. She denied other associated symptoms such as fevers, chills, shortness of breath, or nipple discharge, as well as any new palpable breast masses or previous trauma to the breasts.

**Figure 1:**
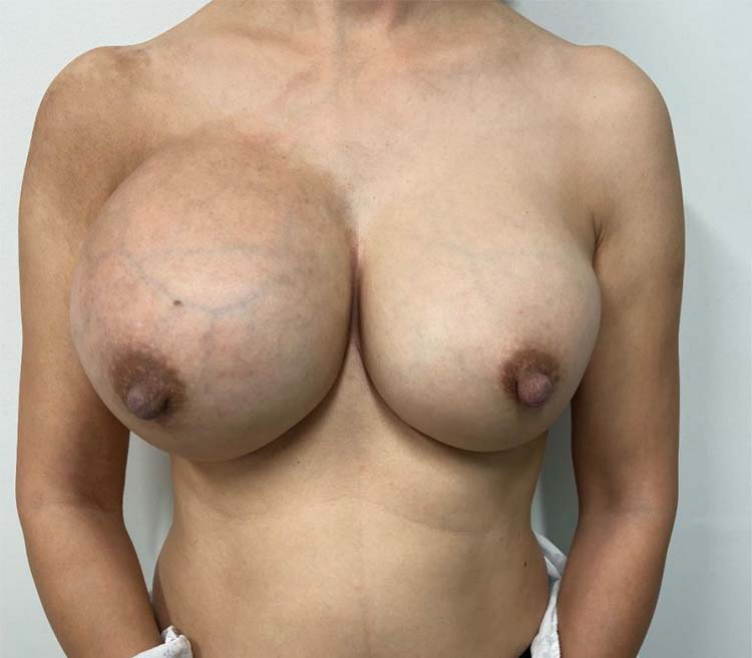
Significant breast asymmetry with right breast being larger and more erythematous than the left breast with accentuation of the superficial veins.

On physical examination, she was afebrile and well-appearing with stable vital signs. The patient had significant breast asymmetry, with the right breast markedly larger than left, as well as associated tenderness to palpation, enlargement of nipple areolar complex and presence of dilated superficial veins **(**Fig. 1**)**. The right breast was noted to be more erythematous and warmer to touch when compared to the left. There were no palpable breast masses, axillary lymphadenopathy or inversion of the nipples.

Diagnostic work up included mammography and magnetic resonance imaging (MRI) of bilateral breasts. Mammography demonstrated presence of breast implants with extensive fibrous capsule discontinuity, suspicious for foci of intracapsular rupture that were more pronounced on the right. There was an air-fluid level within the implant of the right breast, representative of an extracapsular rupture which likely accounted for the size discrepancy between the breasts. MRI demonstrated diffuse nodular thickening of both capsules surrounding the PAAG implants, which was associated with mural nodules with nonvascularized finger-like projections ranging in size from 3 mm to 1.4 cm. Although these findings were benign, the patients acute breast enlargement, pain and evidence of extracapsular rupture with extravasation warranted surgical management.

**Figure 2:**
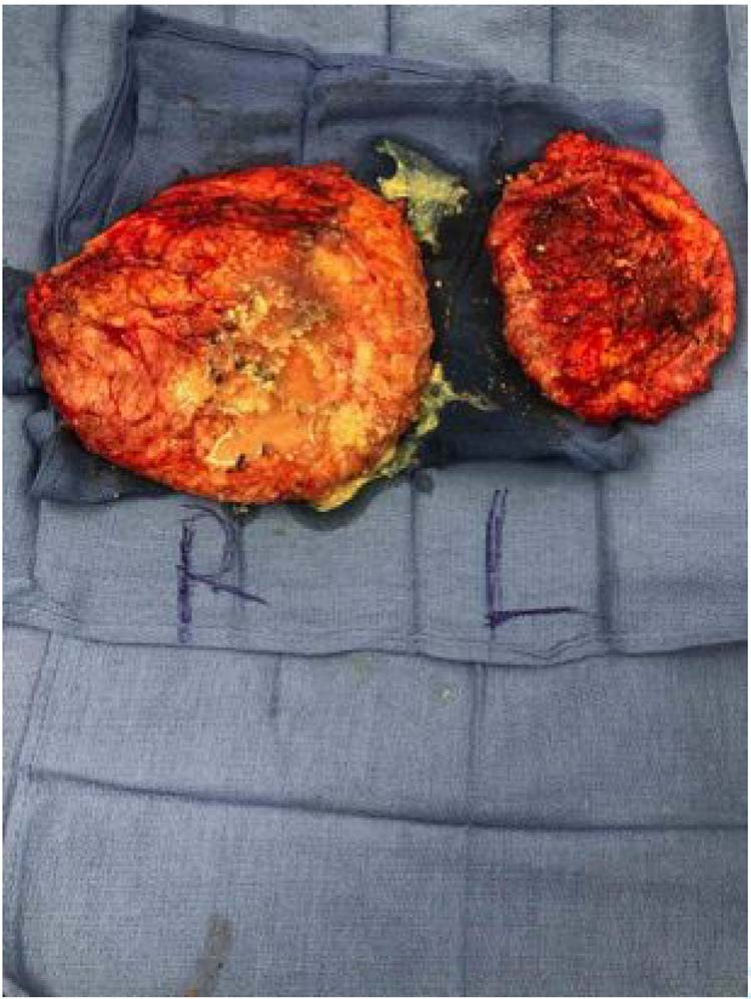
Gross pathological specimen demonstrating extensive fibrotic capsules and porridge-like consistency of PAAG.

The decision was made to proceed with removal of polyacrylamide containing capsules followed by immediate breast reconstruction. Wide inframammary fold incisions were used for completion of nipple-sparing mastectomy and en bloc capsulectomy in order to remove PAAG containing breast capsules as completely as possible. Copious wound irrigation using pulse lavage was performed to minimize presence of polyacrylamide particles. Intraoperative findings demonstrated bilateral breast capsules that had extensive fibrotic scar tissue and were filled with large amounts of porridge-like PAAG with moderate, diffuse infiltration into breast tissue **(**Fig. 2**)**. The capsules were subsequently sent for pathological analysis.

The patient underwent immediate breast reconstruction using prepectoral tissue expander placement utilizing two subcutaneous drains per breast. She had an uncomplicated postoperative course and was discharged home on postoperative Day 1.

**Figure 3:**
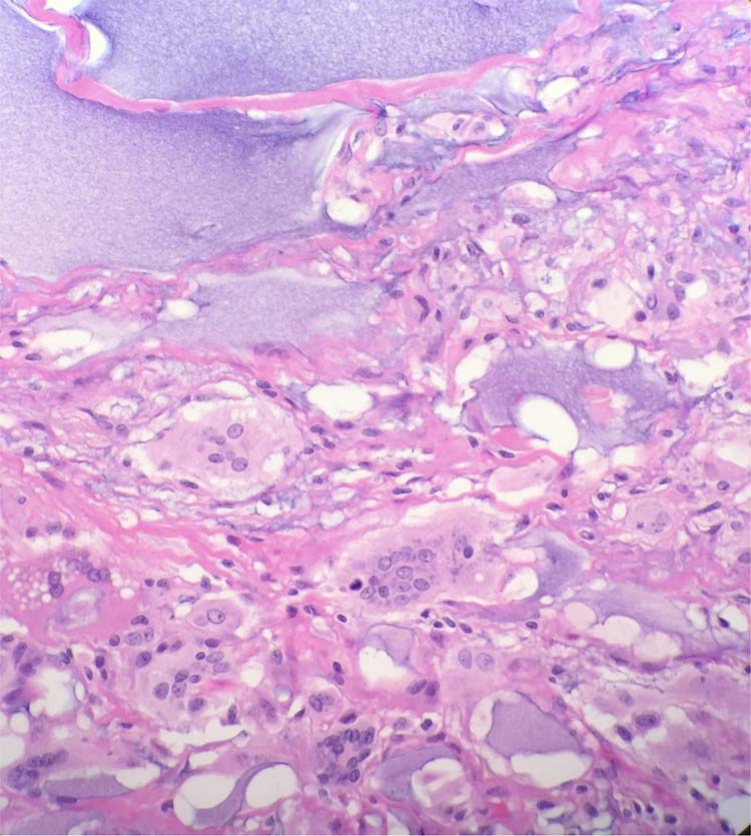
Microscopic examination reveals benign breast tissue with fibrosis, foreign body giant cells and chronic inflammatory cells (H&E 20×) Extensive, scattered, purple, gelatin-like material was also present which represents the presence of polyacrylamide hydrogel.

Gross pathologic examination of breast specimens demonstrated presence of pink-tan colored membranous tissue, consistent with fibrotic capsular tissue intermixed with polymers of polyacrylamide. Microscopic examination demonstrated presence of benign breast tissue with fibrosis, foreign body giant cells and chronic inflammatory cells. There was extensive purple, gelatin-like material which represented the presence of polyacrylamide hydrogel **(**Fig. 3**)**.

## DISCUSSION

The indication for bilateral nipple-sparing mastectomy and en bloc capsulectomy was supported by histopathological analysis of excised breast tissue which demonstrated extensive inflammation and damage at the cellular level. The foreign body reaction and chronic inflammatory infiltrate observed in our patient is in accordance with available literature which suggested the dismal biocompatibility of PAAG [4]. Our histopathological analysis is also consistent with data derived from tissue samples with PAAG by Leung *et al*. which demonstrated extensive foreign body reactions with profound fibrosis and inflammation [5]. Other histological findings have been described by Christensen *et al*., who found evidence of granuloma formation consisting of lymphocytes, foreign body cells, and macrophages surrounding locations of PAAG injection [6].

Several approaches have been suggested in attempt to create guidelines for treatment of complications related to augmentation mammoplasty with PAAG. Conservative management has been extensively evaluated in two notable reviews. Amin *et al*. demonstrated that intralesional steroid injections resulted in resolution of minor PAAG-related inflammatory reactions without recurrence [7]. Qiao *et al*. evaluated the possibility of hydrogel evacuation using aspiration—proving it to be exceedingly difficult with poor efficacy [8].

A study of 325 patients with earlier PAAG-related complications concluded that PAAG evacuation with fibrotic tissue removal and pocket irrigation via periareolar approach was reliable in ensuring adequate removal of PAAG [9]. Additional studies suggested that surgical evacuation and capsulectomy may be required for extensive tissue expansion ‘in order to coagulate any suspected bleeding areas, to debride granulated and necrotic tissue, and to eliminate injected PAAG as much as possible’ [10]. Importantly, however, our case is the first demonstration of a bilateral nipple-sparing mastectomy and en bloc capsulectomy for the successful surgical removal of polyacrylamide 18 years after injection.

In conclusion, complications surrounding PAAG injections have been demonstrated to an extent that they should likely be avoided for augmentation mammoplasty. This case is particularly interesting because it describes unprovoked breast tissue expansion 18 years after PAAG injection. It is important to collect all available data on such cases, as their occurrence in the USA is particularly rare. The authors also aim to highlight the potential for markedly delayed complications after polyacrylamide augmentation mammoplasty, which may require prompt recognition and surgical management, as was done with our patient.

## CONFLICT OF INTEREST STATEMENT

None declared.

## FUNDING

None.
